# Apoptotic cell: linkage of inflammation and wound healing

**DOI:** 10.3389/fphar.2014.00001

**Published:** 2014-01-21

**Authors:** Yu-Sheng Wu, Shiu-Nan Chen

**Affiliations:** College of Life Science, National Taiwan UniversityTaipei, Taiwan

**Keywords:** apoptosis, inflammation, wound healing, cellular physiology, wound repair

## Abstract

We consider that from the wound to the healing process, the physiology point key to linkage of the process is still unclear. The process from inflammation to the wound healing is divided into three phases: (1) inflammation process, (2) tissue formation, and (3) tissue remodeling. The inflammation program includes cell produced related factors and immune cells infiltration. We thought the inflammation factors that may be also involved in the followed healing process. But the question is “what kind of factor is the major key involved in the end of the inflammation then to initiate the healing.” We suspect that the apoptosis of immune cell may be the major key to end of inflammation and to initiate the healing.

## INTRODUCTION

**FIGURE 1:**
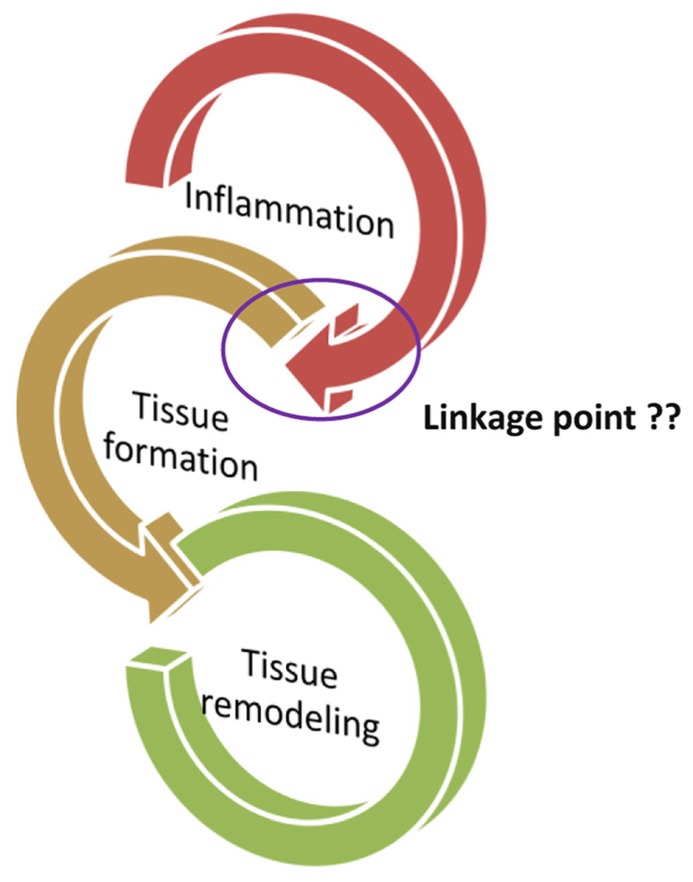
**The process from inflammation to the wound healing is divided into three phases: (1) Inflammation process, (2) tissue formation, and (3) tissue remodeling. ** The important question, “what is the key linkage between the tissue formation and inflammation?”

Wound healing is a complex process involving soluble mediators, blood cells, extracellular matrix, and parenchymal cells (Singer and Clark, 1999; Bullers et al., 2012; Pesce et al., 2013). The process from inflammation to the wound healing is divided into three phases: (1) inflammation process, (2) tissue formation, and (3) tissue remodeling (**Figure 1**; Eming et al., 2007). The inflammatory phase is marked by platelet accumulation, coagulation, and leukocyte migration. The tissue formation is characterized by re-epithelialization, angiogenesis, fibroplasia, and wound contraction. Finally, the remodeling phase takes place over a period of months, during which the dermis responds to injury with the production of collagen and matrix proteins and then returns to its pre-injury phenotype (Kirsner and Eaglstein, 1993; Castillo-Briceno et al., 2011; Bainbridge, 2013). The normal healing response begins the moment the tissue is injured. Peripheral blood components filtrated into the site of injury, the platelets contact with exposed collagen and other elements of the extracellular matrix through the process from inflammation to wound healing (Diegelmann and Evans, 2004). This contact triggers the platelets to release clotting factors as well as essential growth factors and cytokines such as platelet-derived growth factor (PDGF), such as stimulation of DNA synthesis and chemotaxis of fibroblasts moreover smooth muscle cells to induce the production of collagen, glycosaminoglycan, and collagenase by fibroblasts through the wound healing process (Lynch et al., 1987; Price et al., 2004; Tettamanti et al., 2004). Furthermore, PDGF appears to transduce its signal through wound macrophages and may trigger the activation of feedback loops and synthesis of endogenous wound PDGF and other growth factors, thereby enhancing the cascade of tissue repair processes required for a fully healed wound (Pierce et al., 1991). In a normal response to injury, platelet aggregation and degranulation of the earliest events in an inflammatory response trigger the release of numerous inflammatory mediators including transforming growth factor-β (TGF-β) from the granules (Wahl et al., 1989; Tatler and Jenkins, 2012; Christmann et al., 2013). TGF-β is implicated in pathogenic fibrotic conditions in kidney, liver, and lung disease, and in scarring of skin wounds as well (Martin, 1997; Sgonc and Gruber, 2013). Accumulating evidence indicates that TGF-β affects integrin-mediated cell adhesion and migration by regulating the expression of integrins, their ligands and integrin-associated proteins (Margadant and Sonnenberg, 2010). Research indicated that a synthetic TGF-β is effective in accelerating wound healing and reducing scarring in pig skin burn, pig skin excision, and rabbit skin excision injury models (Huang et al., 2002).

## INFLAMMATION PROCESS

Inflammation is known to be a crucial adaptive response for animals, and the mechanism is a complex interaction with molecular mediators even the functions of immune cells in a microenvironment through a response that occurs at all levels of biological organization (Allavena et al., 2008). In this process, cooperation among cells and mediators occurs, and a wide range of factors are involved in the classical immune response, including the stage of the inflammation process; the tissue or organ involved; and whether the inflammation is acute and resolving or chronic and non-resolving (Punchard et al., 2004). The inflammation process involves vascular permeability, active migration of blood cells, and the passage of plasma constituents into injurious tissue (Maslinska and Gajewski, 1998). Through the infiltration of immune cells, studies have shown that the inflammation process plays a crucial role in atherosclerosis (Sbarsi et al., 2007). Blood leukocytes, mediators of host defenses and inflammation, localize in the earliest lesions of atherosclerosis in experimental animals. The study of inflammation in atherosclerosis has afforded considerable new insight into the mechanisms underlying the recruitment of leukocytes (Libby et al., 2002). Recently, studies have indicated the role of inflammation in Alzheimer’s disease (AD; Schott and Revesz, 2013). Inflammatory components related to AD neuroinflammation include brain cells, such as microglia and astrocytes, the complement system, and cytokines and chemokines (Rubio-Perez and Morillas-Ruiz, 2012). Regarding cancer development (Gregory, 2013), pro-inflammatory cytokines, including interleukin (IL)-1α, IL-1β, IL-6, IL-8, IL-18, chemokines, matrix metallopeptidase (MMP)-9, and vascular endothelial growth factor (VEGF) are primarily regulated by the transcription factor nuclear factor (NF)-kB, which is active in most tumors and is induced by carcinogens (Aggarwal et al., 2006). Cutaneous wound repair is a tightly regulated and dynamic process involving blood clotting, inflammation, formation of new tissue, and tissue remodeling (Muller et al., 2012). Thrombin is the protease involved in blood coagulation. Its deregulation can lead to hemostatic abnormalities, which range from subtle subclinical to serious life-threatening coagulopathies, i.e., during septicemia (Danckwardt et al., 2013). Inflammation and blood coagulation are the part of the innate host protection mechanism on vascular injury, infection or other wound. Not only the cell of the innate immune system but also activated endothelial cells and platelets are actively involved in acute and chronic inflammation: they release of pro-inflammatory mediators, expose adhesion molecules and receptors, proteases and its inhibitors, clotting factors and associated proteins, and recruit leukocytes (Strukova, 2006). In the process, PAR family serve as sensors of serine proteinases of the blood clotting system in the target cells involved in inflammation. Activation of PAR-1 by thrombin and of PAR-2 by factor leads to a rapid expression and exposure on the membrane of endothelial cells of both adhesive proteins that mediate an acute inflammatory reaction and of the tissue factor that initiates the blood coagulation cascade.(Dugina et al., 2002).

## MEDIATORS OF INFLAMMATION

In the inflammation, microenvironment is related to cellular trans-differentiation, migration, proliferation, survival, and extracellular matrix formation. The growth factors likely to be involved are PDGF, TNF-α and TNF-β, HGF, TGF-β2, epidermal growth factor (EGF), and fibroblast growth factor (FGF). Cytokines such as IL-1, IL-6, IL-8, IL-10, and interferon gamma (INF-γ) are also thought to play a role (Morescalchi et al., 2013). It is clearly a balance between appropriate fibroblast activation and the fibrosis that results from their continuing activation. Multiple growth factors have been implicated in fibroblast migration and activation, but much attention has been recently focused on the PDGF family of growth factors and their cognate receptors (PDGFRs; Nemenoff, 2012). Research has documented that PDGF exerts autocrine, mitogenic effect on keratinocytes to support epidermal proliferation and stabilization of the dermoepidermal junction during wound closure. In addition, it stimulates vessel maturation by recruitment and differentiation of pericytes to the immature-endothelial channels (Hellberg et al., 2010).

Studies have investigated the cytokines involved in the inflammation response by using various animal models. The expression of pro-inflammatory cytokines, such as tumor necrosis factor alpha (TNF)-α, is significantly increased in the adipocytes of obese animals (ob/ob mouse, db/db mouse, and fa/fa Zucker rat; Hotamisligil et al., 1993). The activation of TNF-α might induce leukocytes express adhesion molecules on the cell surface (Dunne et al., 2003; Bruderer et al., 2013; Li et al., 2013), leading to diapedesis through individual vascular endothelial cells (Carman and Springer, 2004). IL-6 is an adipokine (Fried et al., 1998) thought to be a mediator of inflammation (Xing et al., 1998; Deng et al., 2012; Tang et al., 2012) that is produced by adipose tissue and liver-resident macrophages that are activated in response to hepatocyte death (Sakurai et al., 2008). IL-6-deficient mice exhibit a marked decrease in inflammatory response, granulation tissue formation, and re-epithelialization (Gallucci et al., 2000). The IL-1 family, which includes IL-1α and IL-1β, exhibits strong pro-inflammatory activities and plays a major role in host responses to exogenous and endogenous noxious stimuli (Gabay et al., 2010). IL-1 induces the expression of adhesion molecules on endothelial cells and elicits stromal cells to release chemokines that promote the recruitment of inflammatory cells at the inflammation site (Dinarello, 1996; Chang et al., 2012; Hu et al., 2012). Such inflammation occurs significantly in cases of comorbidity and might contribute to the increased risk of developing cardiovascular accidents observed in these patients (Carpagnano et al., 2010). IL-10, a cytokine with anti-inflammatory properties, plays a central role in infection that involves limiting the immune response to pathogens and thereby preventing damage to the host (Saraiva and O’Garra, 2010). Recently, research has shown that IL-10 and related cytokines can facilitate the tissue-healing process in injuries caused by infection or inflammation (Ouyang et al., 2011). According to these studies, mediators thought to be involved in the regulation of inflammation responses such as leukocyte recruitment, adhesion molecule expression, and wound healing in the late phase of inflammation.

## IMMUNE CELL-WOUND HEALING

Immune cells are involved in virtually every aspect of the wound repair process, from the initial stages where they participate in hemostasis and work to prevent infection to later stages where they drive scar formation (Wilgus, 2008). Evidence supporting a central role for T lymphocytes in the control of wound healing is provided by studies which examine the *in vivo* effects of alternate forms of T cell manipulation on various parameters of healing (Barbul and Regan, 1990) and Neutrophils as important to wound healing as they help control infection, however, they also release harmful enzymes which damage healthy tissue surrounding the wound site (Brubaker et al., 2011). Investigations have enumerated many of the specific proteins that are produced by wound macrophages at the site of injury. These include the following: (1) chemoattractants that recruit and activate additional macrophages at the site of injury, (2) growth factors that promote cellular proliferation and protein synthesis, (3) proteases and extra-cellular matrix molecules, and (4) factors that may restrain tissue growth once repair is completed (DiPietro, 1995). Neutrophils arrive first within a few minutes, followed by monocytes and lymphocytes. They produce a wide variety of proteinases and reactive oxygen species as a defense against contaminating microorganisms, and they are involved in the phagocytosis of cell debris. Neutrophil play a role as primarily phagocytosis appearing approximately 24 h after injury and contribute to decreasing the infection in the wound. Neutrophils are not paramount to the process of wound healing or collagen synthesis (Park and Barbul, 2004). Research has been shown a role of neutrophil in wound healing for the production of neutrophil growth factors, such as granulocyte/macrophage colony-stimulating factor (GM-CSF; Canturk et al., 2001). Experiments with cultures of keratinocytes established from ^-^^/^^-^ and ^+^^/^^+^ mice revealed a retardation in wound closure in CXCR2 ^-^^/^^-^keratinocytes, role for this receptor on keratinocytes in epithelial resurfacing that is independent of neutrophil recruitment (Devalaraja et al., 2000). In the resolution and regeneration stages, macrophages appear to remove large cell debris as well as apoptotic neutrophils, the key scavengers for resolving inflammation and facilitating tissue regrowth, furthermore, experiment illustrated that the depletion of macrophages in zebrafish model leads to the delay of the clearance of cell debris, decrease of regeneration speed, and formation of vacuoles in the regenerating fin (Li et al., 2012). Recently, research has shown that wound healing requires a coordinated interplay among cells, growth factors, and extracellular matrix proteins. Central to this process is the endogenous mesenchymal stem cell (MSC), which coordinates the repair response by recruiting other host cells and secreting growth factors and matrix proteins. MSCs are self-renewing multipotent stem cells that can differentiate into various lineages of mesenchymal origin such as bone, cartilage, tendon, and fat (Maxson et al., 2012).

## APOPTOTIC CELL-WOUND HEALING

**FIGURE 2:**
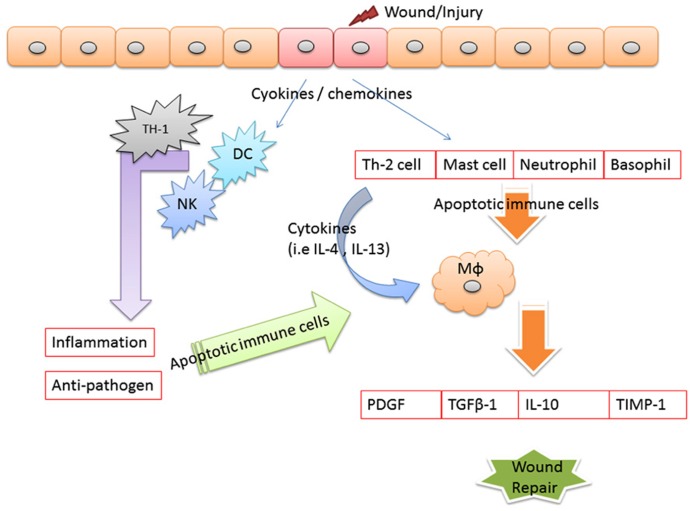
**Apoptotic immune cell linkage wound healing.** When tissues are damaged, inflammatory mediators are released. Where macrophages and become activated by various cytokines, such as interferon-γ (IFN-γ), that are released from neighboring inflammatory cells, including neutrophils, natural killer (NK) cells, resident tissue macrophages, and T cells. In the end of the inflammation, we can observe the apoptosis of the immune cells and the apoptotic cells cleared by macrophages. We thought that clearance by macrophages of cells apoptosis is a key point phenomenon associated with actively tissue formation from wound inflammation. The apoptotic immune may drive the conversion of the immune response into a wound healing response, which is characterized by the accumulation of macrophages that promote wound healing and fibrosis through the production of MMPs (including MMP12, tissue inhibitor of metalloproteinases 1 (TIMPs1), growth factors [including platelet-derived growth factor (PDGF)], and cytokines [such as transforming growth factor-β1 (TGF-β1)].

Evidence illustrated that apoptosis is involved in the resolution of various phases of tissue repair. In the early phases of tissue repair, inflammatory cells underwent apoptosis starting as early as 12 h after wound injured (Brown et al., 1997). Examined apoptotic patterns in cells in open wounds created in rats, found that apoptosis marked observed in the inflammatory cells of the scab. In this research found that apoptosis in myofibroblasts initiated on day 12, peaked at day 20, and resolved at day 60. These findings suggest that myofibroblast apoptosis initiated about the same time at the end of the wound following to the healing (Desmouliere et al., 1995). Stromal keratocyte apoptosis has been well-characterized as an early initiating event of the corneal wound healing response, triggering subsequent cellular processes that include bone marrow-derived cell infiltration, proliferation, and migration of residual keratocyte cells, and, in some circumstances, generation of myofibroblast cells (Wilson et al., 2007). Impaired phagocytosis of apoptotic neutrophils by Vav3^-^^/^^-^ (guanine-nucleotide exchange factors implicated in leukocyte functions by relaying signals from immune response receptors and integrins to Rho-GTPases) macrophages was causal for their reduced release of active TGF-β1, for decreased myofibroblasts differentiation and myofibroblast-driven wound contraction to cause the situation of delayed wound healing (Sindrilaru et al., 2009). Apoptotic cells released growth signals that stimulated the proliferation of progenitor or stem cells by caspases 3 and 7 proteases which involves the caspase-mediated activation of phospholipase A2 and the subsequent production and release of the lipid signal prostaglandin E2, a stimulator of cell proliferation and mice lacking either of these caspases were deficient in skin wound healing (Li et al., 2010). We thought the inflammation factors that may be also involved in the followed healing process. But the question is “what kind of factor is the major key involved in the end of the inflammation then to initiate the healing.” We suspect that the apoptosis of immune cell may be the major key to end of inflammation and to initiate the healing as shown in **Figure 2**.

## CONCLUSION

We hypothesized that the key point to end of the inflammation is the apoptotic activity of immune cells. Apoptosis is considered a vital component of various processes including normal cell turnover, proper development and functioning of the immune system, hormone-dependent atrophy, embryonic development, and chemical-induced cell death (Elmore, 2007). In the inflammation response, the mediators induce the infiltration of activated immune cells into inflammation site to protect the tissue against the pathogen infection. In the end of the inflammation, we can observe the apoptosis of the immune cells and the apoptotic cells cleared by macrophages. We thought that clearance by macrophages of cells apoptosis is a key point phenomenon associated with actively tissue formation from wound inflammation.

## Conflict of Interest Statement

The authors declare that the research was conducted in the absence of any commercial or financial relationships that could be construed as a potential conflict of interest.

## References

[B1] Aggarwal B. B. Shishodia S. Sandur S. K. Pandey M. K. Sethi G. (2006). Inflammation and cancer: how hot is the link? *Biochem. Pharmacol*. 72 1605–1621 10.1016/j.bcp.2006.06.029 16889756

[B2] Allavena P. Sica A. Solinas G. Porta C. Mantovani A. (2008). The inflammatory micro-environment in tumor progression: the role of tumor-associated macrophages. *Crit. Rev. Oncol. Hematol.* 66 1–9 10.1016/j.critrevonc.2007.07.004 17913510

[B3] Bainbridge P. (2013). Wound healing and the role of fibroblasts. *J. Wound Care* 22 407–412 23924840 10.12968/jowc.2013.22.8.407

[B4] Barbul A. Regan M. C. (1990). The regulatory role of lymphocytes-T in wound-healing. *J. Trauma-Injury Infect. Crit. Care* 30 S97–S100 10.1097/00005373-199012001-00021 2254999

[B5] Brown D. L. Kao W. W. Y. Greenhalgh D. G. (1997). Apoptosis down-regulates inflammation under the advancing epithelial wound edge: Delayed patterns in diabetes and improvement with topical growth factors. *Surgery* 121 372–380 10.1016/S0039-6060(97)90306-8 9122866

[B6] Brubaker A. L. Schneider D. F. Kovacs E. J. (2011). Neutrophils and natural killer T cells as negative regulators of wound healing. *Expert. Rev. Dermatol.* 6 5–8 10.1586/edm.10.66 21442028 PMC3063646

[B7] Bruderer M. Alini M. Stoddart M. J. (2013). Role of HOXA9 and VEZF1 in endothelial biology. *J. Vasc. Res.* 50 265–278 10.1159/000353287 23921720

[B8] Bullers S. Berry H. Ingham E. Southgate J. (2012). The resolution of inflammation during the regeneration of biological scaffolds by human tissue. *J. Tissue Eng. Regen. Med.* 6 218–218

[B9] Canturk N. Z. Esen N. Vural B. Canturk Z. Kirkali G. Oktay G. (2001). The relationship between neutrophils and incisional wound healing. *Skin Pharmacol. Appl. Skin Physiol.* 14 108–116 10.1159/000056340 11316969

[B10] Carman C. V. Springer T. A. (2004). A transmigratory cup in leukocyte diapedesis both through individual vascular endothelial cells and between them. *J. Cell Biol.* 167 377–388 10.1083/jcb.200404129 15504916 PMC2172560

[B11] Carpagnano G. E. Spanevello A. Sabato R. Depalo A. Palladino G. P. Bergantino L. (2010). Systemic and airway inflammation in sleep apnea and obesity: the role of ICAM-1 and IL-8. *Transl. Res.* 155 35–43 10.1016/j.trsl.2009.09.004 20004360

[B12] Castillo-Briceno P. Bihan D. Nilges M. Hamaia S. Meseguer J. Garcia-Ayala A. (2011). A role for specific collagen motifs during wound healing and inflammatory response of fibroblasts in the teleost fish gilthead seabream. *Mol. Immunol.* 48 826–834 10.1016/j.molimm.2010.12.004 21232799 PMC3048961

[B13] Chang M. C. Lin L. D. Zwei-Ching Chang J. Huang C. F. Chuang F. H. Lee J. J. (2012). Regulation of vascular cell adhesion molecule-1 in dental pulp cells by interleukin-1beta: the role of prostanoids. *J. Endod.* 38 774–779 10.1016/j.joen.2012.02.030 22595111

[B14] Christmann R. B. Sampaio-Barros P. Stifano G. Borges C. L. De Carvalho C. R. Kairalla R. (2013). Key roles for interferon- and TGF-beta-regulated genes, and macrophage activation in progressive lung fibrosis associated with Systemic Sclerosis. *Arthritis Rheum*. 10.1002/art.38288 [Epub ahead of print] PMC4439004 24574232

[B15] Danckwardt S. Hentze M. W. Kulozik A. E. (2013). Pathologies at the nexus of blood coagulation and inflammation: thrombin in hemostasis, cancer, and beyond. *J. Mol. Med.* 91 1257–1271 10.1007/s00109-013-1074-5 23955016 PMC3825489

[B16] Deng J. Wang X. R. Qian F. Vogel S. Xiao L. Ranjan R. (2012). Protective role of reactive oxygen species in endotoxin-induced lung inflammation through modulation of IL-10 expression. *J. Immunol.* 188 5734–5740 10.4049/jimmunol.1101323 22547702 PMC3358534

[B17] Desmouliere A. Redard M. Darby I. Gabbiani G. (1995). Apoptosis mediates the decrease in cellularity during the transition between granulation-tissue and scar. *Am. J. Pathol.* 146 56–66 7856739 PMC1870783

[B18] Devalaraja R. M. Nanney L. B. Qian Q. H. Du J. G. Yu Y. C. Devalaraja M. N. (2000). Delayed wound healing in CXCR2 knockout mice. *J. Invest. Dermatol.* 115 234–244 10.1046/j.1523-1747.2000.00034.x 10951241 PMC2664868

[B19] Diegelmann R. F. Evans M. C. (2004). Wound healing: an overview of acute, fibrotic and delayed healing. *Front. Biosci. *9 283–289 10.2741/1184 14766366

[B20] Dinarello C. A. (1996). Biologic basis for interleukin-1 in disease. *Blood* 87 2095–2147 8630372

[B21] DiPietro L. A. (1995). Wound healing: the role of the macrophage and other immune cells. *Shock* 4 233–240 10.1097/00024382-199510000-00001 8564549

[B22] Dugina T. N. Kiseleva E. V. Chistov I. V. Umarova B. A. Strukova S. M. (2002). Receptors of the PAR-family as a link between blood coagulation and inflammation. *Biochemistry (Mosc.)* 67 65–74 10.1023/A:1013952114485 11841341

[B23] Dunne J. L. Collins R. G. Beaudet A. L. Ballantyne C. M. Ley K. (2003). Mac-1, but not LFA-1, uses intercellular adhesion molecule-1 to mediate slow leukocyte rolling in TNF-alpha-induced inflammation. *J. Immunol.* 171 6105–6111 14634125 10.4049/jimmunol.171.11.6105

[B24] Elmore S. (2007). Apoptosis: a review of programmed cell death. *Toxicol. Pathol.* 35 495–516 10.1080/01926230701320337 17562483 PMC2117903

[B25] Eming S. A. Krieg T. Davidson J. M. (2007). Inflammation in wound repair: molecular and cellular mechanisms. *J. Invest. Dermatol.* 127 514–525 10.1038/sj.jid.5700701 17299434

[B26] Fried S. K. Bunkin D. A. Greenberg A. S. (1998). Omental and subcutaneous adipose tissues of obese subjects release interleukin-6: Depot difference and regulation by glucocorticoid. *J. Clin. Endocrinol. Metab.* 83 847–850 10.1210/jc.83.3.847 9506738

[B27] Gabay C. Lamacchia C. Palmer G. (2010). IL-1 pathways in inflammation and human diseases. *Nat. Rev. Rheumatol.* 6 232–241 10.1038/nrrheum.2010.4 20177398

[B28] Gallucci R. M. Simeonova P. P. Matheson J. M. Kommineni C. Guriel J. L. Sugawara T. (2000). Impaired cutaneous wound healing in interleukin-6-deficient and immunosuppressed mice. *FASEB J.* 14 2525–2531 10.1096/fj.00-0073com 11099471

[B29] Gregory C. D. (2013). Inflammation and cancer revisited: an hypothesis on the oncogenic potential of the apoptotic tumor cell. *Autoimmunity* 46 312–316 10.3109/08916934.2012.755961 23320865

[B30] Hellberg C. Ostman A. Heldin C. H. (2010). PDGF and vessel maturation. *Recent Results Cancer Res.* 180 103–114 10.1007/978-3-540-78281-0_7 20033380

[B31] Hotamisligil G. S. Shargill N. S. Spiegelman B. M. (1993). Adipose expression of tumor necrosis factor-alpha – direct role in obesity-linked insulin resistance. *Science* 259 87–91 10.1126/science.7678183 7678183

[B32] Huang J. S. Wang Y. H. Ling T. Y. Chuang S. S. Johnson F. E. Huang S. S. (2002). Synthetic TGF-beta antagonist accelerates wound healing and reduces scarring. *FASEB J.* 16 1269–1270 12153996 10.1096/fj.02-0103fje

[B33] Hu W. Xia L. J. Chen F. H. Wu F. R. Tang J. Chen C. Z. (2012). Recombinant human endostatin inhibits adjuvant arthritis by down-regulating VEGF expression and suppression of TNF-alpha, IL-1beta production. *Inflamm. Res.* 61 827–835 10.1007/s00011-012-0477-z 22610149

[B34] Kirsner R. S. Eaglstein W. H. (1993). The Wound-Healing Process. *Dermatol. Clin.* 11 629–640 8222347

[B35] Li A. L. Yang Y. Y. Gao C. Lu J. Y. Jeong H. W. Liu B. H. (2013). A SALL4/MLL/HOXA9 pathway in murine and human myeloid leukemogenesis. *J. Clin. Invest.* 123 4195–4207 10.1172/JCI62891 24051379 PMC3784519

[B36] Li F. Huang Q. Chen J. Peng Y. L. Roop D. R. Bedford J. S. (2010). Apoptotic cells activate the “Phoenix Rising” pathway to promote wound healing and tissue regeneration. *Sci. Signal*. 3: ra13 10.1126/scisignal.2000634 PMC2905599 20179271

[B37] Li L. Yan B. Shi Y. Q. Zhang W. Q. Wen Z. L. (2012). Live imaging reveals differing roles of macrophages and neutrophils during zebrafish tail fin regeneration. *J. Biol. Chem.* 287 25353–25360 10.1074/jbc.M112.349126 22573321 PMC3408142

[B38] Libby P. Ridker P. M. Maseri A. (2002). Inflammation and atherosclerosis. *Circulation* 105 1135–1143 10.1161/hc0902.104353 11877368

[B39] Lynch S. E. Nixon J. C. Colvin R. B. Antoniades H. N. (1987). Role of platelet-derived growth-factor in wound-healing – synergistic effects with other growth-factors. *Proc. Natl. Acad. Sci. U.S.A.* 84 7696–7700 10.1073/pnas.84.21.7696 3499612 PMC299367

[B40] Margadant C. Sonnenberg A. (2010). Integrin-TGF-beta crosstalk in fibrosis, cancer and wound healing. *EMBO Rep.* 11 97–105 10.1038/embor.2009.276 20075988 PMC2828749

[B41] Martin P. (1997). Wound healing – aiming for perfect skin regeneration. *Science* 276 75–81 10.1126/science.276.5309.75 9082989

[B42] Maslinska D. Gajewski M. (1998). Some aspects of the inflammatory process. *Folia Neuropathol.* 36 199–204 10079600

[B43] Maxson S. Lopez E. A. Yoo D. Danilkovitch-Miagkova A. Leroux M. A. (2012). Concise review: role of mesenchymal stem cells in wound repair. *Stem Cells Transl. Med.* 1 142–149 10.5966/sctm.2011-0018 23197761 PMC3659685

[B44] Morescalchi F. Duse S. Gambicorti E. Romano M. R. Costagliola C. Semeraro F. (2013). Proliferative vitreoretinopathy after eye injuries: an overexpression of growth factors and cytokines leading to a retinal keloid. *Mediators Inflamm*. 2013: 269787 10.1155/2013/269787 PMC3806231 24198445

[B45] Muller A. K. Meyer M. Werner S. (2012). The roles of receptor tyrosine kinases and their ligands in the wound repair process. *Semin. Cell Dev. Biol.* 23 963–970 10.1016/j.semcdb.2012.09.015 23059791

[B46] Nemenoff R. (2012). Wound healing: a role for HDACs in inhibition of fibroblast proliferation through repression of PDGF receptor-alpha. Focus on “Repression of PDGF-R-alpha after cellular injury involves TNF-alpha, formation of a c-Fos-YY1 complex, and negative regulation by HDAC”. *Am. J. Physiol.* 302 C1588–C1589 10.1152/ajpcell.00095.2012 22460709

[B47] Ouyang W. J. Rutz S. Crellin N. K. Valdez P. A. Hymowitz S. G. (2011). Regulation and functions of the IL-10 family of cytokines in inflammation and disease. *Annu. Rev. Immunol.* 29 71–109 10.1146/annurev-immunol-031210-101312 21166540

[B48] Park J. E. Barbul A. (2004). Understanding the role of immune regulation in wound healing. *Am. J. Surg.* 187 11S–16S 10.1016/S0002-9610(03)00296-4 15147986

[B49] Pesce M. Patruno A. Speranza L. Reale M. (2013). Extremely low frequency electromagnetic field and wound healing: implication of cytokines as biological mediators. *Eur. Cytokine Netw.* 24 1–10 10.1684/ecn.2013.0332 23674517

[B50] Pierce G. F. Mustoe T. A. Altrock B. W. Deuel T. F. Thomason A. (1991). Role of platelet-derived growth-factor in wound-healing. *J. Cell. Biochem.* 45 319–326 10.1002/jcb.240450403 2045423

[B51] Price R. D. Das-Gupta V. Harris P. A. Leigh I. M. Navsaria H. A. (2004). The role of allogenic fibroblasts in an acute wound healing model. *Plast. Reconstr. Surg.* 113 1719–1729 10.1097/01.PRS.0000117367.86893.CE 15114134

[B52] Punchard N. A. Whelan C. J. Adcock I. (2004). The journal of inflammation. *J. Inflamm. (Lond.)* 1: 1 10.1186/1476-9255-1-1 PMC1074343 15813979

[B53] Rubio-Perez J. M. Morillas-Ruiz J. M. (2012). A review: inflammatory process in Alzheimer’s disease, role of cytokines. *ScientificWorldJournal* 2012: 756357 10.1100/2012/756357 PMC3330269 22566778

[B54] Sakurai T. He G. Matsuzawa A. Yu G. Y. Maeda S. Hardiman G. (2008). Hepatocyte necrosis induced by oxidative stress and IL-1 alpha release mediate carcinogen-induced compensatory proliferation and liver tumorigenesis. *Cancer Cell* 14 156–165 10.1016/j.ccr.2008.06.016 18691550 PMC2707922

[B55] Saraiva M O’Garra A. (2010). The regulation of IL-10 production by immune cells. *Nat. Rev. Immunol.* 10 170–181 10.1038/nri2711 20154735

[B56] Sbarsi I. Falcone C. Boiocchi C. Campo I. Zorzetto M. De Silvestri A. (2007). Inflammation and atherosclerosis: The role of TNF and TNF receptors polymorphisms in coronary artery disease. *Int. J. Immunopathol. Pharmacol.* 20 145–154 17346438 10.1177/039463200702000117

[B57] Schott J. M. Revesz T. (2013). Inflammation in Alzheimer’s disease: insights from immunotherapy. *Brain* 136 2654–2656 10.1093/brain/awt231 23983027

[B58] Sgonc R. Gruber J. (2013). Age-related aspects of cutaneous wound healing: a mini-review. *Gerontology* 59 159–164 10.1159/000342344 23108154

[B59] Sindrilaru A. Peters T. Schymeinsky J. Oreshkova T. Wang H. L. Gompf A. (2009). Wound healing defect of Vav3(-/-) mice due to impaired beta(2)-integrin-dependent macrophage phagocytosis of apoptotic neutrophils. *Blood* 113 5266–5276 10.1182/blood-2008-07-166702 19147786

[B60] Singer A. J Clark R. a. F. (1999). Mechanisms of disease – cutaneous wound healing. *N. Engl. J. Med.* 341 738–746 10.1056/NEJM199909023411006 10471461

[B61] Strukova S. (2006). Blood coagulation-dependent inflammation. Coagulation-dependent inflammation and inflammation-dependent thrombosis. *Front. Biosci.* 11: 59–80 10.2741/1780 16146714

[B62] Tang E. H. C. Libby P. Vanhoutte P. M. Xu A. M. (2012). Anti-inflammation therapy by activation of prostaglandin EP4 receptor in cardiovascular and other inflammatory diseases. *J. Cardiovasc. Pharmacol.* 59 116–123 10.1097/FJC.0b013e3182244a12 21697732 PMC3191244

[B63] Tatler A. L. Jenkins G. (2012). TGF-beta activation and lung fibrosis. *Proc. Am. Thorac. Soc.* 9 130–136 10.1513/pats.201201-003AW 22802287

[B64] Tettamanti G. Grimaldi A. Rinaldi L. Arnaboldi F. Congiu T. Valvassori R. (2004). The multifunctional role of fibroblasts during wound healing in *Hirudo medicinalis* (Annelida, Hirudinea). *Biol. Cell* 96 443–455 10.1016/j.biolcel.2004.04.008 15325073

[B65] Wahl S. M. Mccartneyfrancis N. Mergenhagen S. E. (1989). Inflammatory and immunomodulatory roles of TGF-beta. *Immunol. Today* 10 258–261 10.1016/0167-5699(89)90136-9 2478145

[B66] Wilgus T. A. (2008). Immune cells in the healing skin wound: influential players at each stage of repair. *Pharmacol. Res.* 58 112–116 10.1016/j.phrs.2008.07.009 18723091

[B67] Wilson S. E. Chaurasia S. S. Medeiros F. W. (2007). Apoptosis in the initiation, modulation and termination of the corneal wound healing response. *Exp. Eye Res.* 85 305–311 10.1016/j.exer.2007.06.009 17655845 PMC2039895

[B68] Xing Z. Gauldie J. Cox G. Baumann H. Jordana M. Lei X. F. (1998). IL-6 is an antiinflammatory cytokine required for controlling local or systemic acute inflammatory responses. *J. Clin. Invest.* 101 311–320 10.1172/JCI1368 9435302 PMC508569

